# Study of jelly drying cashew apples (*Anacardium occidentale L*.) processing

**DOI:** 10.1002/fsn3.2565

**Published:** 2021-11-05

**Authors:** Tan Phat Dao, Duc Ngoc Vu, Duong Vu Nguyen, Van Thinh Pham, Thi Yen Nhi Tran

**Affiliations:** ^1^ Institute of Environmental Sciences Nguyen Tat Thanh University Ho Chi Minh City Vietnam; ^2^ Faculty of Environmental and Food Engineering Nguyen Tat Thanh University Ho Chi Minh City Vietnam; ^3^ Graduate University of Science and Technology Vietnam Academy of Science and Technology Ha Noi Vietnam; ^4^ Department of Chemical and Food Technology Nong Lam University Ho Chi Minh City Vietnam; ^5^ Faculty of Food Science and Technology Ho Chi Minh City University of Food Industry Ho Chi Minh City Vietnam

**Keywords:** blanching, cashew apples (*Anacardium occidentale L*.), drying, jelly, osmosis

## Abstract

Cashew apples, a by‐product accrued during the manufacture of cashew nuts, have abundant nutritional values but are not widely utilized due to the presence of substances that cause acrid taste. In this study, we attempted the production of a dried jelly cashew apple product and optimized three main processing stages including blanching, osmotic, and drying. The results showed suitable conditions at 6 mm thickness in the blanching process. The osmotic process recorded a temperature of 35°C, within 1.5 h, the ratio of sugar syrup/ingredient 2:1 with sugar syrup 60 Bx, and the addition of 0.6% citric acid on the total weight of ingredients and 0.02% CaCl_2_. The drying process at 55°C within 267 min had the highest ascorbic acid content (TAA), total phenolic content (TPC), and content of tannin compounds (TTC) retention. These parameters refer to a product that has good organoleptic acceptability in terms of taste, acrid content, and a high ability to retain major nutrients. Furthermore, the product recovery efficiency is 21.45%. Jelly drying cashew apples (JDC) are formed to help take advantage of by‐products, contributing to adding value for the cashew industry.

## Graphical abstract

We successfully researched the technological process of jelly production from cashew apples, which was previously a by‐product of acrid substances, named tannin. Sensory value and good nutrient retention are achieved through blanching, osmosis, and drying.

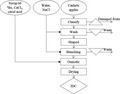

## 1 INTRODUCTION

Cashew (*Anacardium ocidental* Linne) is a tropical tree, often grows wild in coastal sandy beaches and natural forests, has strong vitality in harsh conditions, and is a staple cash crop in many regions in South Asia countries. The fruit of cashew, commonly called cashew apples, is usually 8–10 times larger than the cashew nut and has been shown to possess antioxidant activity that is around 5 times higher than that of orange, and 12 times higher than pineapple (Attri, 2009; Talasila et al., 2012). In addition, cashew apples also have high triterpenoid and phenolic content, which plays an important role in inhibiting bacteria causing gastritis in humans (Salehi et al., 2020). Nutrition‐wise, cashew apples contain around 80%–90% of juice (pH 3.9‒4.1), in which sugar accounts for about 8%‒10% and consists of mainly reducing sugar, vitamins C, B1, B2, PP, and carotenoids (Abreu et al., 2013). Cashew apples are also abundant in minerals including iron (30 times higher than lemon), calcium, and phosphorus and contain other nutrients in high quantities including about 9% protein, 4% lipid, 8% cellulose, and 1% pectin (Das & Arora, 2017). However, the high content of tannin (around 0.2%‒0.5%) is responsible for the acrid and bitter taste of the fruit, discouraging its consumption and thus causing difficulties in processing into palatable products (Assis et al., 2007; Emmanuelle et al., 2016). Therefore, cashew apple is often discarded after harvest or used as fertilizers, narrowing the economic value of the cashew industry and contributing to the accumulation of agricultural waste.

The processing of cashew apple into a wide array of food products such as juice, wine, cashew vinegar, and jam has been attempted previously (Lavinas et al., 2006). However, those processes have been shown to be unable to completely eliminate the residual tannin in the fruit juice, leaving a moderate taste of acrid and bitterness and negatively affecting the consumer acceptance of the resulting product. In a recent study, we successfully implemented the blanching process with saline solution to significantly reduce the tannin in the cashew apple while still maintaining adequate vitamin C content in the product (Dao et al., 2021). In the current study, a manufacture process in which cashew apples are processed into jelly dried cashew apple (JDC) was proposed. First, we investigated the blanching process with regard to changes in ascorbic acid content (TAA), total phenolic content (TPC), and total tannin content (TTC) during blanching (Dao et al., 2021). The blanching process aims to inactivate the browning enzyme, increase moisture loss, and reduce the tannin content to an acceptable organoleptic level. Following that, we evaluated the effect of temperature and time on TAA, TPC, and TTC in the flavoring permeation process induced by syrup. Finally, the obtained products were dried, and drying parameters including temperature and drying time were optimized based on the change of TAA, TPC, TTC, color, and moisture content of the resulting product. The results are expected to contribute to the diversification of products derived from cashew and to the reduction of agricultural waste.

## 2 MATERIALS AND METHODS

### 2.1 Samples

Cashews were harvested in Binh Phuoc Province (Coordinates 11^o^45'N 106^o^55'E), Vietnam, in January 2021. Most of the selected cashew apples were yellow. The fruit was de‐nutted and stored at 0°C for further use.

### 2.2 Chemicals and agent

The chemicals used such as Folin‐Ciocalteu, gallic acid, and KMnO_4_ were purchased from Sigma‐Aldrich. Dichlorophenolindophenol, denoted as DCPIP (purity 99.7%), HCl (purity 36.5%), and Na_2_CO_3_ (purity 99.5%) were purchased from China. Sugar (99.8%), citric acid, CaCl_2_, and E211 were purchased from Vietnam.

### 2.3 JDC processing

**FIGURE 1:**
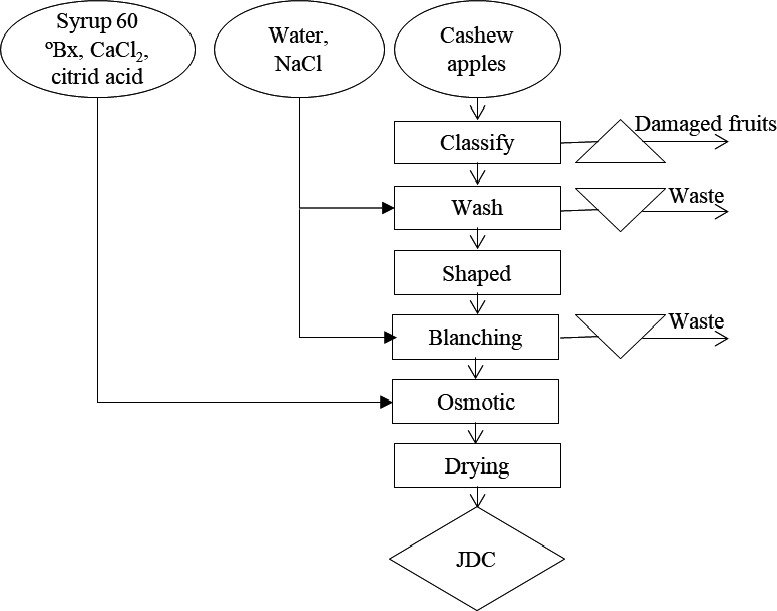
Steps of technology to create JDC

Cashews after harvest were de‐seeded and sorted to remove damaged fruits. The height of the fruit ranged from 4 to 5 cm, and the fruit radius ranged from 1.5 to 2.5 cm. Cashew apples were washed and subjected to blanching, osmosis, and drying processes (Figure 1).

The blanching process was carried out under previously reported optimal conditions including 1% saline solution at 70°C for 3 min (Dao et al., 2021). The material size was allowed to vary from 4 to 10 mm. Following that, the osmosis process was performed by using 60 Bx syrup with a ratio between materials and syrup solution of 1:2. The solution was added with 0.6% citric acid to neutralize the taste and 0.2% CaCl_2_ to create hardness. The temperature was allowed to range from 35°C to 65°C and the time from 1 to 2.5 h. Then, the obtained cashew apples were dried at a temperature of 55°C–65°C to obtain JDC product (moisture 12% ± 2%).

### 2.4 Determination of total ascorbic acid

The content of ascorbic acid was determined according to the method of AOAC 967.21, as previously attempted on cashews by Dao et al. (2021); Tran et al., 2020). First, 1 g of sample was extracted three times and then titrated to 100 ml with distilled water. Then, 10 ml of sample solution was added to 1 ml of 0.04% HCl, followed by titration with DCPIP. The control sample was carried out in the same way as above, and the analytical sample was diluted with 0.1 g of ascorbic acid up to 100 ml. The assays were repeated 3 times and titrated from colorless solution to pale pink within 30 s. The volume of DCPIP solution was recorded.

### 2.5 Determination of total tannin content

To determine the tannin content, the method of Lowenthal is used according to the description of the Dao et al. (2021). First, 10g of raw materials is extracted 3 times and added with distilled water to 100 ml. After that, 10 ml of extracted solution is added to the 250‐ml Erlen flask, followed by addition of 1 ml indigo carmine and 100 ml distilled water. The mixture was titrated with KMnO_4_ solution until yellow color appears. Similarly, the blank is performed with a similar step in the above but the extract was replaced with distilled water.

### 2.6 Determination of phenolic content

The content of total phenolics is determined by Folin‐Ciocalteu colorimetric method (Dao et al., 2021; Pham et al., 2020). First, 1 g samples are extracted with distilled water and refer to nearest volume. The collected filtrate (0.5 ml) was transferred into a dark tube, followed by addition of 2 ml Folin‐Ciocalteu reagent (diluted 10 times with distilled water) and 2.5 ml sodium carbonate solution (20% w/v). The mixture was then incubated in the dark for 1 h before being measured photometrically at an absorption wavelength of 765 nm.

## 3 RESULT AND DISCUSSION

### 3.1 Effect of conditions during blanching

Appropriate blanching conditions can maintain the material structure and nutritional quality for cashew apples while reducing the content of tannin. In this experiment, cashew apples were blanched under the following conditions: blanching temperature of 70°C, blanching solution of 1% NaCl, and for 2 min. In this condition, the vitamin C and polyphenol content reached 0.50 ± 0.02 mg/g (22% reduction), 4.69 ± 0.55 mg/g (38% reduction), respectively, and the tannin content decreased by 55% compared to that of the dry samples (Dao et al., 2021). We varied the width of the cross‐sectional slices of cashew apples to find out how material size could affect the main nutritional content of the product. The upper and lower size ranges (4 and 10 mm) have been selected to ensure the quality of the subsequent cashew apple jelly product and to fully appreciate the influence of the parameters during the blanching process.

**FIGURE 2:**
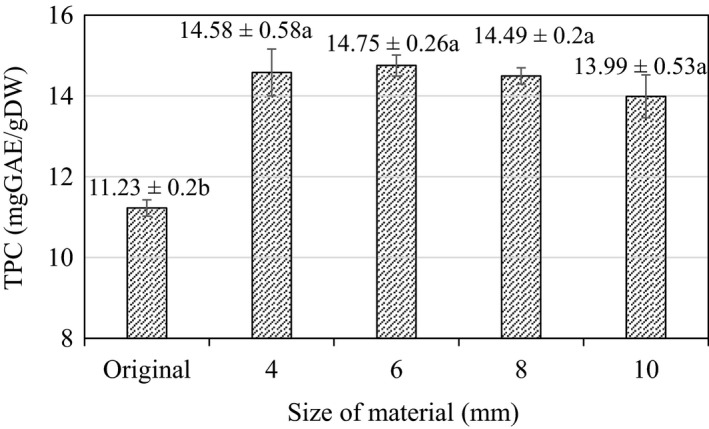
Total polyphenol content in the process of blanching at different sizes. Here, Original is the value of the initial material (raw material). (a–b) indicate a statistically significant difference (*p* < .05) between different slice thicknesses

The TPC with respect to different sizes of cashew apple slices during blanching is shown in Figure 2. The highest polyphenol content (14.75 ± 0.26 mgGAE/gDW) corresponds to the size of 6 mm, and the lowest TPC was measured in the control sample, which has not been heat‐treated (11.23 ± 0.2 mgGAE/gDW). According to the results of statistical analysis, it was found that the material thickness ranging 4–10 mm seemed to have no significant effect on TPC (*p* > .05). However, there was a clear difference of TPC (around 23%) between the original unheated cashew slices with blanched samples.

The insignificant difference in TPC content at different thicknesses can be explained by negligible differences in contact area between samples. Therefore, heat transfer and water penetration rates would be all similar across samples. Figure 2 shows that browning enzyme inactivation can occur under all conditions and that the material size did not seem to affect TPC. In addition, the increase in TPC during blanching may be attributed to the reduction of enzyme‐mediated polyphenol degradation (complete inactivation of native polyphenol oxidase) and the release of bound phenolic acids from the breakdown of cellular components of the plant cell walls in the leafy vegetable (Francisco et al., 2010). This initial increase in the TPC of the plant correlates with the report of Dewanto et al. (2002) where they reported that cooling or blanching could increase phenol contents in vegetables. Bamidele et al. (2017); Bamidele et al. (2017) also reported in a similar study on the increase in polyphenol content of green leafy vegetables during blanching.

**FIGURE 3:**
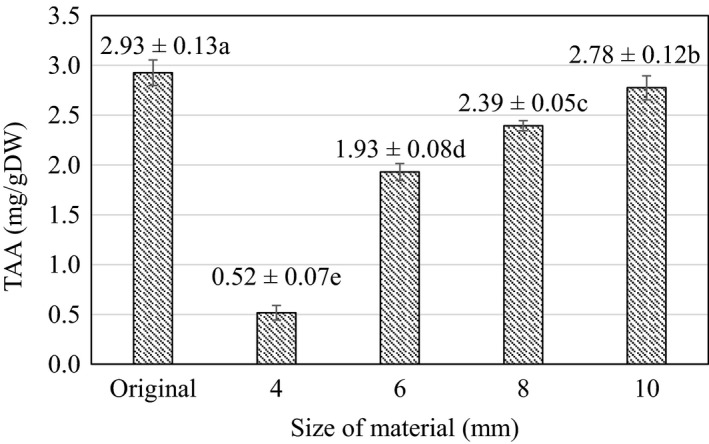
Ascorbic acid content during blanching at different sizes. Here, Original is the value of the initial material (raw material). (a–e) indicate a statistically significant difference (*p* < .05) between different slice thicknesses

TAA content in cashew apple slices during blanching at different sizes is shown in Figure 3. According to the results of statistical analysis, it was found that varying the material thickness from 4 to 10 mm had a significant effect on the TAA content (*p* < .05) during the blanching process.

The TAA content increased from 0.52 ± 0.07 to 2.78 ± 0.12 mg/gDW when increasing the material size from 4 mm to 10 mm. To be specific, at material size of 4 mm, TAA value reached 0.52 ± 0.07 mg/gDW and then improved to 2.39 ± 0.05 mg/gDW at material size of 8 mm, which is only about 18.17% lower than TAA of the control sample (without heat treatment). At 10 mm, there was no difference in TAA of the blanched samples compared to the original sample. This could be explained by the difference in material thickness, leading to discrepancy in the rate of heat transfer from the environment to the center of the cashew slice. Therefore, ascorbic acid in the 4 mm cashew slice decomposed more quickly than the thicker slice. Indeed, it has been shown that ascorbic acid is unstable and could be easily destroyed by many factors such as pH, temperature, light, oxygen, and enzymes (Gupta et al., 2008). In cashew apples, TAA is an important criterion to evaluate the quality of the product. Therefore, the size that allows for maximum retention of TAA was selected for subsequent experiments.

**FIGURE 4:**
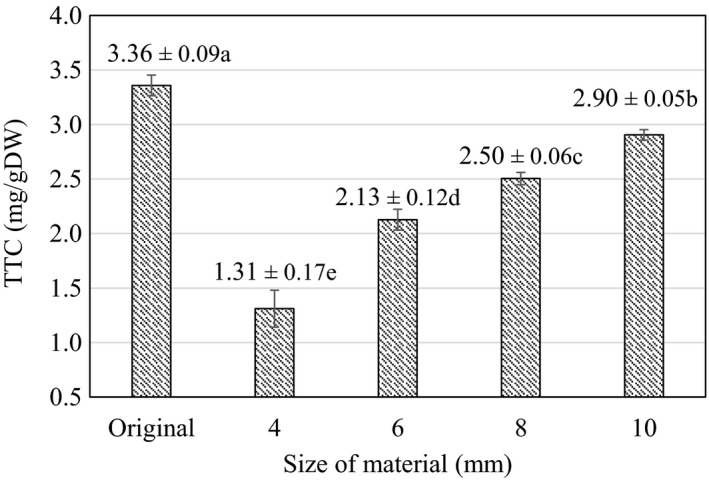
Total tannin content during blanching at different sizes. Here, Original is the value of the initial material (raw material). (a–e) indicate a statistically significant difference (*p* < .05) between different slice thicknesses

The TTC recorded in cashew apple slices during blanching at different sizes is shown in Figure 4. The reduction of TTC is considered as an important criterion in converting this by‐product source into edible products. According to the results of statistical analysis, it was found that the blanching process significantly reduced the TTC content (*p* < .05) in cashew apple slices at all thicknesses.

TTC value increased from 1.31 ± 0.17 to 2.9 ± 0.05 mg/gDW when increasing the material thickness from 4 mm to 10 mm. Material thickness of 4 mm corresponded to TTC value of 1.31 ± 0.17 mg/gDW, which is a 60.98% reduction compared to that of the control. TTC reached 2.13 ± 0.1 mg/gDW at material size of 6 mm and decreased to 2.5 ± 0.06 mg/gDW at material size of 8mm. TTC peaked at 2.9 ± 0.05 mg/gDW, at material size of 10 mm, which is a 13.52% reduction compared to that of the control sample. The results are explained by the properties of tannins that dissolve and hydrolyze in water, especially hot water. At the same time, tannins could also hydrolyze to form gallic acid and some other polyols under the influence of heat transfer from external to the inside of the cashew fruit—which explains the increase in tannins at different blanching sizes (Sieniawska & Baj, 2017). Besides, due to the polar nature of tannins, part of TTC is dissolved in water under blanching at elevated temperatures (Dao et al., 2021). This causes the TTC to tend to increase linearly with the material thickness under blanching.

The tannin content represents an important factor in converting by‐products such as cashew apples into food. Compared to the whole unblanched cashew apple, blanching and cutting the material to the thickness of 4 mm seemed to lower the tannin content (1.31 ± 0.17 mg/gDW) by about 60.98%. Similarly, the TTC figure at 6 mm (2.13 ± 0.1 mg/gDW) is about 36.67% lower. At 4–6 mm sizes, the tannins–acrid substances are well removed and the materials showed appropriate visual value to be used in subsequent processing stages. Besides, in order to retain the main nutritional value of samples, the size of 6 mm was selected as the most suitable. At this material size, the TAA value reached 1.93 ± 0.08 mg/gDW (65.95% retention), and TPC reached 14.75 ± 0.26 mgGAE/gDW, an increase of about 23.91% compared to the control sample. For those reasons, 6 mm was selected for processing in the next stage. This result is also similar to the study of Olalusi & Erinle (2019) on *Anacardium Occidentale* L. in Nigeria, which showed that cashew apples were sliced at 7 mm before acid pretreatment and dried at 55°C, the tannin content decreased with a large value from 2.2028 g compared to 261.3 g in fresh samples, and other contents such as vitamin C and calcium obtained good values. Vitamin C is the main ingredient in cashew apple, as reported by Lagnika et al. (2019); vitamin C content was 2.9 ± 0.74 mg/100 g; polyphenols reached 1.05 ± 0.37 mg/ml during blanching of cashew apples; this result is quite low when compared with TAA and TPC of cashew apples evaluated in this study. The difference in TPC and TAA values may be due to differences in cultivation, environmental conditions, and age of plants (Nguyen et al., 2020).

### 3.2 Effect of conditions on osmosis

During the osmosis process, sugar is an important ingredient that creates the taste of the product. In addition, sugar also has the role of inhibiting the growth of microorganisms, prolonging the storage time. The temperature and time of osmosis are the main factors affecting the quality of cashew apple jelly. In this experiment, the effects of time and temperature on total dissolved solids, TPC, TAA, and TTC of the cashew fruit slices was evaluated. A sugar solution with TSS value of 60 Bx was fitted for the experiments, and the sugar solution (>60% w/w) might allow faster penetration as recommended earlier by Najafi et al. (2014).

**FIGURE 5:**
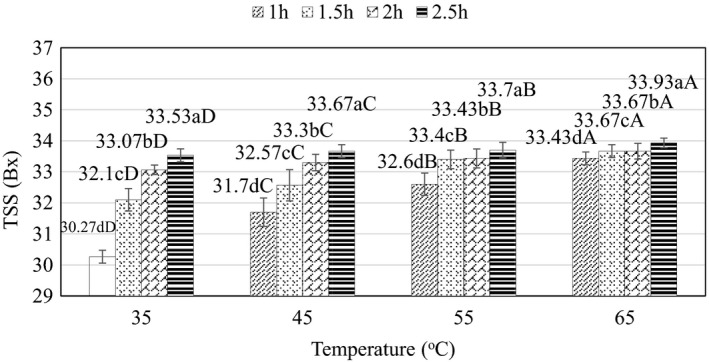
Effect of temperature and time on TSS (Bx) during osmosis. (a–d) and (A–D) indicate a statistically significant difference (*p* < .05) between temperature and time at different points

The influence of temperature and osmosis time on total dissolved solids (Bx) is shown in Figure 5. According to the analysis results, TSS values recorded in the raw materials after osmosis time at different temperatures were all significantly different (*p* < .05). Increasing the temperature from 35°C to 65°C leads to an increase of TSS; however, at 35°C the rate of increase of TSS is slower than that at 65°C and at 65°C and higher there is no change in TSS in raw materials. The results show that at the temperature of 65°C and time of 2.5 h, TSS was the highest (33.93 ± 0.15 Bx). The lowest TSS was recorded at 35°C and duration of 1 h (30.27 ± 0.21 Bx). Indeed, due to slower permeation rate at low temperatures, a slower increase in TSS is more often observed in the material at low temperatures than at high temperatures. Previously, a temperature of 35°C was used in the permeation of *Hylocereus polyrhizusis* in the study of Najafi et al. (2014). The results showed that at this condition the increase in sugar level and time made the product more susceptible to dehydration than the untreated sample. The influence of TSS on permeation was also reported by Sereno et al. (2001), showing that an increase in time and temperature increases the permeation of sucrose into the feedstock, and the rate of dehydration is higher with prolongation of these conditions.

**FIGURE 6:**
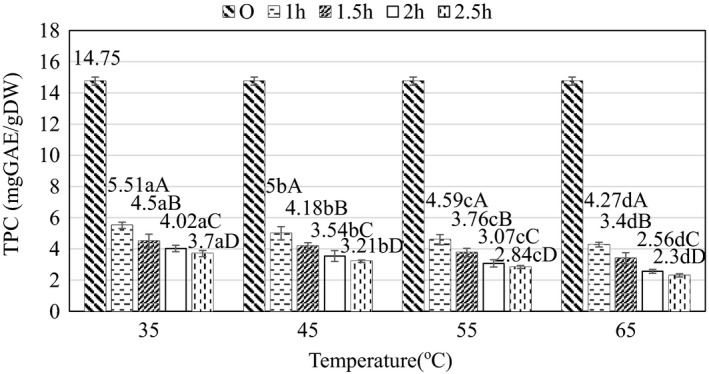
Effect of time and temperature on total polyphenol content during osmosis. Here, Original (O) is the value of the initial material (raw material). (a–d) and (A–D) indicate a statistically significant difference (*p* < .05) between temperature and time at different points

The change of TPC value during osmosis is shown in Figure 6. TPC values are statistically different at different time points and temperatures at the 95% confidence level (*p* < .05). At the same time, the factors of temperature and time, according to the results of statistical analysis, interact with each other (*p* < .05).

According to Figure 6, when increasing the osmosis time, the total polyphenol content in the product sample tends to decrease; this decrease is shown notably in the first hour (from 11.06 to 5.51 mgGAE/gDW at 35°C) and then decreased slowly in the period 1–2.5 h. In terms of both the interaction of temperature and osmosis time, at 35°C and time of 1 h, the highest TPC was maintained (5.51 ± 0.2 mgGAE/gDW) and the lowest TPC was recorded at 65°C and time of 2.5 h (2.3 ± 0.1 mgGAE/gDW). Temperature and time seemed to correlate with each other when evaluating the objective function as TPC. As the osmosis is prolonged, the polyphenol content in the sample tends to escape to the outside environment as well as participate in chemical and biochemical reactions such as decomposition reaction, oxidation reaction, and hydrolysis reaction to reduce the amount of polyphenols in the raw materials (Blanda et al., 2009). This result is consistent with the report of Devic et al. (2010) showing that increasing the apple osmotic temperature to 45°C–60°C decreased TPC content significantly. At the same time, when the temperature increased, the distance between cells is also increased, leading to faster osmosis rate and more TPC loss to the environment. Therefore, the use of the lowest temperature and time is necessary in order to maximize the retention of these total polyphenols.

**FIGURE 7:**
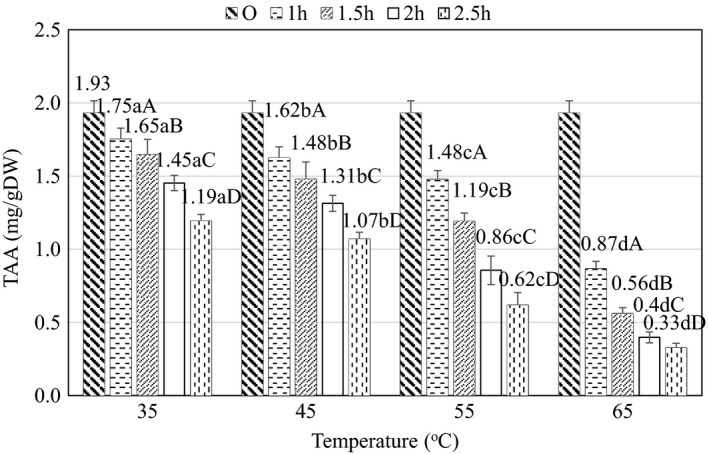
Effect of temperature and time on ascorbic acid content during osmosis. Here, Original (O) is the value of the initial material (raw material). (a– d) and (A–D) indicate a statistically significant difference (*p* < .05) between temperature and time at different points

The change of TAA value during osmosis is shown in Figure 7. According to the results of statistical analysis, the TAA content at the time and temperature levels is statistically different at 95% confidence level (*p* < .05) and the temperature and there is an interaction between time and temperature (*p* < .05).

According to Figure 7, as the osmotic time increased from 1 to 2.5 h, the TAA content decreased, but in the first 1 h at 35°C there was no statistically significant difference between TAA values (*p* > .05). At the same time, when increasing the temperature from 35°C to 65°C, the rate of decrease in TAA content increased and decreased sharply in the first 1–1.5 h. On the other hand, at 55°C–65°C, there was a sharp decrease in TAA compared to the temperature from 35°C to 45°C. Indeed, the higher temperature might lower the TAA content, in turn accelerating the rate of decomposition and oxidation. At the same time, when the temperature increased from 45°C to 65°C, the amount of TAA decreased significantly because thermal decomposition of vitamin C accelerated underexposure of osmotic temperature of higher than 45°C, as previously indicated (Devic et al., 2010). Furthermore, it is possible that some of the ascorbic acid migrated from the raw material to the osmotic solution, as shown in the study of Argandoña et al. (2010) on the reduction of ascorbic acid during pepper permeation.

**FIGURE 8:**
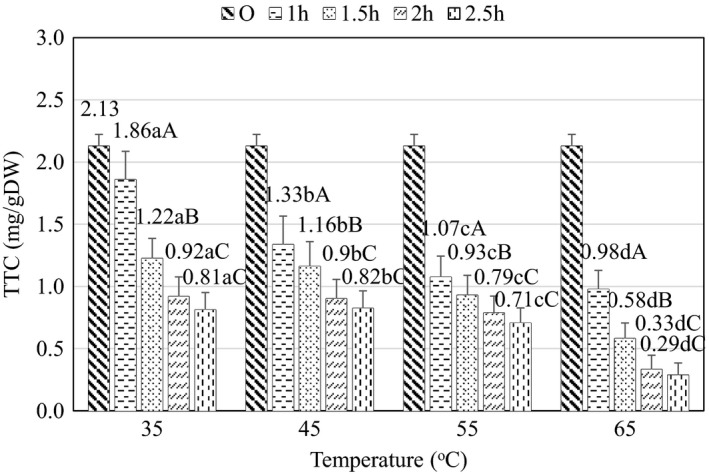
Effect of temperature and time on total tannin content during osmosis. Here, Original (O) is the value of the initial material (raw material). (a–d) and (A– C) indicate a statistically significant difference (*p* < .05) between temperature and time at different points

The change of TTC value during osmosis is shown in Figure 8. According to the results of statistical analysis, TTC contents at different time and temperature levels are statistically different at the 95% confidence level (*p* < .05), and the temperature and time factor have an interaction with each other (*p* < .05).

The results also indicate that the osmotic temperature seemed to positively relate to TTC, which is consistent with the temperature survey results of Dao et al. (2021). On the other hand, it can be observed in Figure 8 that the osmotic time from 1 to 2 h exerted a significant decrease in TTC, but when prolonging the duration from 2 to 2.5 h, TTC did not seem to decline further. This result can be explained by the high osmosis rate in the period from 1 to 2 h due to the large difference in concentrations of solutes in the osmotic medium and raw materials. By contrast, in the period from 2 to 2.5 h, the difference in the concentration of solutes of the raw materials and the medium is closer to the equilibrium, so the osmosis rate decreases, which means that the rate of dissolution and release of tannins into the environment decrease. At the same time, when the temperature increases, the cells expand, facilitating the movement of the osmotic process to the equilibrium and thus more tannins are released to the environment (Deshpande et al., 1986).

Texture is considered as an important criterion to evaluate samples during osmosis. The differences in appearance of cashew apples are shown in Table 1. Generally, after 2 h of osmosis at different temperatures, the structure of cashew apples is soft and the outer skin is wrinkled.

**TABLE 1:** Appearance values of cashew apple samples at different osmotic temperatures

Osmosis	35°C	45°C	55°C	65°C
1.0 h	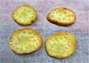	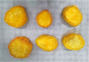	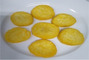	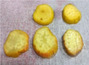
Description	Acrid taste, little sweetness, light brown in color, strong smell, dried fruit flesh	Acrid taste, sweet, light color, not too harsh, strong smell, soft fruit flesh	Less acrid, strong sweetness, not too harsh light color, very soft flesh	Less acrid, strong sweetness, harsh, light color, very soft flesh
1.5 h	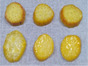	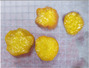	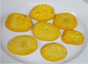	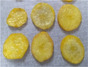
Description	Less acrid, medium sweetness, not too harsh, light color, characteristic aroma, flexible fruit flesh	Less acrid, sweet, not too harsh, light color, soft flesh, characteristic aroma, wrinkled outer skin	Less acrid, strong sweetness, harsh, light color, very soft flesh, characteristic aroma, wrinkled outer skin	Less acrid, strong sweetness, harsh, very soft flesh, light color, characteristic aroma, wrinkled outer skin
2.0 h	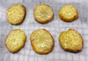	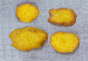	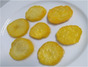	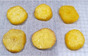
Description	Less acrid, sweet, harsh, dark color, characteristic aroma	Less acrid, sweet, harsh, light color, soft flesh, characteristic aroma, wrinkled outer skin	Less acrid, strong sweetness, harsh, light color, very soft flesh, characteristic aroma, wrinkled outer skin	Less acrid, strong sweetness, harsh, very soft flesh, light color, characteristic aroma, wrinkled outer skin
2.5 h	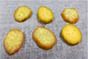	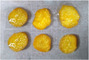	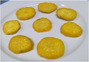	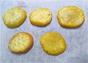
Description	Less acrid, strong sweetness, harsh, dark color, characteristic aroma	Less acrid, strong sweetness, harsh light color, soft flesh, characteristic aroma, wrinkled outer skin	Less acrid, strong sweetness, light color, very soft flesh, characteristic aroma, wrinkled outer skin	Less acrid, strong sweetness, harsh, very soft flesh, light color, characteristic aroma, wrinkled outer skin

During the osmosis process, samples treated at 35°C and 45°C for 1.5 h showed similar nutritional values as well as product taste with TSS in the range of 32 BX. TPC, TAA, and TCC values of the two products have no statistical difference. However, in terms of product appearance, the sample at 35°C was more consistent in terms of sweetness and plasticity than the products obtained with 45°C blanching. Indeed, the time of 1.5 h and the temperature of 35°C gave a TSS of about 32.1 ± 0.36 Bx. The product had a medium, but not too harsh, sweet taste and retained the optimal TAA of about 1.65 ± 0.1 mg/gDW, which is a reduction of 14.68% compared to the unblanched sample (1.93 ± 0.08 mg/gDW). The product also had TPC of about 4.5 ± 0.4 mgGAE/gDW, reduced by 69.5% compared to that after blanching (14.75 ± 0.26 mgGAE/gDW). TTC value was also significantly reduced to 1.22 ± 0,16 mg/gDW reduced by 42.49% compared to after blanching 2.13 ± 0.1 mg/gDW, while retaining the characteristic mild acrid taste of the product. This is consistent with previous results showing that in the osmosis process, moderate temperature (45°C) is nutritionally beneficial and minimizes losses total ascorbic acid content (Devic et al., 2010).

### 3.3 Effect of drying process

#### 3.3.1 Effect of drying temperature on TAA and TPC

**FIGURE 9:**
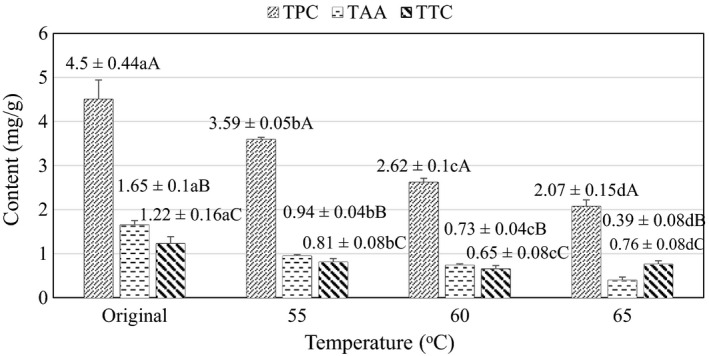
Effect of drying temperature on TAA, TPC, and TTC. Here, Original is the value of the initial material (raw material). (a–d) indicate a statistically significant difference in TAA (*p* < .05) between different drying temperatures. A, B, C means are dependent on TPC, TAA, and TTC columns, respectively

Temperature is an important factor in the drying process of raw materials. Temperature affects loss of biological activity, TAA, TPC, TTC, and the taste of the product as well as production efficiency. Experimental results on biological activity with respect to drying temperature are shown in Figure 9.

Figure 9 shows the influence of drying temperature on the content of TAA and TPC. According to the results, the concentrations of TAA and TPC at different temperatures were statistically different at the 95% confidence level (*p* < .05). Observed from the graph, it can be seen that temperature and biological activity are negatively correlated, which is consistent with the results of previous studies (Nhi et al., 2020). The highest content of polyphenols can be seen at the drying temperature of 55°C (3.59 ± 0.05 mgGAE/gDW), a decrease of 20.24% compared to the post‐permeation sample (4.50 ± 0.44 mgGAE/gDW). The highest TAA (0.94 ± 0.04 mg/gDW) was recorded in the sample dried at 55°C, which is a 42.88% reduction compared with TAA of the control, permeated sample (1.65 ± 0.1 mg/gDW). The effects of temperature were similarly observed in the study of Dimoso et al. (2020) on the solar heating and drying process of cashew slices. However, the TAA in the product only reached 0.73–0.85 g/100g which represents a lower nutritional content compared to current study. Besides, the loss of biological activities may be due to the influence of high temperature, prolonged exposure to the air, which accelerates decomposition and oxidation of TPC and TAA (Johnston et al., 2006; Tran et al., 2020).

The result of Figure 9 shows that, when increasing the drying temperature from 55°C to 65°C, TTC showed no clear changes. However, the samples after osmosis showed decreased TTC compared with the original sample. This result is explained by the combination of moisture and drying temperature of the raw materials, which makes the hydrolyzed TTC to oxidize or to form complexes with other ingredients such as proteins more easily, causing TTC to reduce when subjected to drying for a long time at high temperature. The TTC after drying decreased by about 34.1% compared to that after osmosis. The decline is also in line with previous results published by Deshpande et al. (1986); Dimoso et al. (2020). TTC values are not significantly different when changing the survey temperature from 55°C to 65°C, this may be due to the loss of unstable tannins in the previous stages, and the remaining tannins in the drying stage are not hydrolyzed, but mostly condensed tannins, which are thermally stable and decomposed at 210°C–215°C (Deshpande et al., 1986).

#### 3.3.2 Effect of temperature to drying time and color

The drying process reduces the moisture content of the raw materials, which plays an important role in increasing the shelf life of products with high moisture content and preserving agricultural products. Therefore, in many agricultural countries, large quantities of food products are dried to increase shelf life, reduce packaging costs, lower volume, retain original flavor, and maintain nutritional value.

Drying times were measured at 55°C, 60°C, and 65°C until the moisture content reached 12%–13%, which is a common condition for jelly products. From Table 2, it is shown that when the drying temperature increases, the drying time also decreases. At 55°C, the drying time lasts about 268.77 min, but when it is increased to 65°C, the remaining time is only about 180.17 min. It can be seen that there is a positive correlation of temperature and drying time to the product. This is consistent with the principle of the drying process: the higher the temperature, the faster the heat transfer of the hot air agent into the material will be. Therefore, the moisture content on the surface of the drying material evaporates faster than at low temperature; however, this factor is detrimental in terms of biological activity as previously mentioned.

**TABLE 2:** Product drying time at different temperatures

Drying temperature (^o^C)	55	60	65
Drying time (min)	268.77 ± 17.35	207.67 ± 11.24	180.17 ± 9.11

Table 3 presents the L* a* b* value of the product at the investigated drying temperatures. The results show that there is a difference in color at the three levels of drying surveyed. Higher drying temperature seemed to induce the darker the color of the dried product, reducing the product's sensory properties (Nguyen et al., 2019).

**TABLE 3:** Colors of products during drying at different temperatures

Drying temperature (^o^C)	Image	L*	a*	b*
55	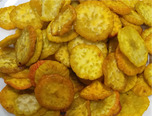	77.46 ± 2.13	−1.48 ± 1.38	38.64 ± 4.04
60	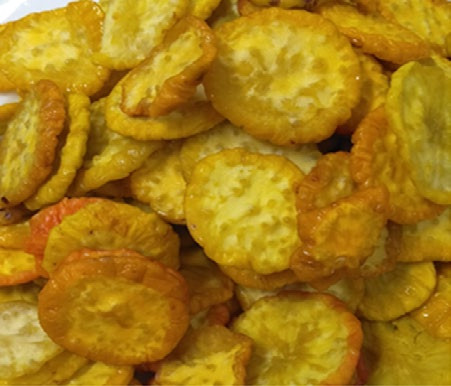	72.58 ± 0.76	2.70 ± 1.83	51.00 ± 6.05
65	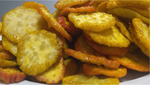	69.52 ± 0.45	1.9 ± 0.788	47.37 ± 3.68

In addition, when increasing the drying temperature from 55°C to 65°C, the product's brightness gradually decreases from 77.46 ± 2.13 to 69.52 ± 0.45. The color of the product is yellow light at drying temperature of 55°C. At 60°C the product color turned into a darker yellow, more inclined towards red. At 65°C the color is darker than the color observed at 60°C. The cause may be due to the oxidation of phenolics under the influence of oxygen to form undesirable color pigments (Prathapan et al., 2009).

The product obtained at the temperature of 55°C seems to retain the optimal TAA and TPC. TPC value retained 79.76%, TAA retained 57.12%, and TTC value decreased 34.1% compared with the control samples which were not heat‐treated. In addition, the average drying time is about 268 min to achieve the desired moisture level (12%–13%). Previous studies (Olalusi & Erinle, 2019) also mentioned the use of a temperature of 55°C for the drying process of cashew apples. At this temperature, the loss of vitamin C can be reduced, the browning effect is less, and the loss of tannin is high. Indeed, the higher the drying temperature can shorten the drying time; however, the product do not retain the color as well as the essential nutrient content which is important in food products especially by‐products such as cashew apples.

The technological process of making jelly from cashew apples was carried out so that the tannin content is reduced while still maintaining acceptable visual value with the maximum nutrient content such as vitamin C and polyphenols. In addition, the yield of cashew apples jelly product was also investigated. The experimental parameter is based on the determination of the sample weight through the optimal point of the blanching, osmosis, and drying processes evaluated previously. The process parameters are shown as follows:

Input materials (1560.75g) ‐> blanching process (859.51 ± 32.65g) ‐> osmosis process (1017.30 ± 0.81g) ‐> drying process (334.73 ± 2.21g).

The production efficiency of cashew apples jelly products reached 21.45% with essential nutritional values such as TPC of 3.59 ± 0.05 mgGAE/gDW, TAA of 0.94 ± 0.04 mg/gDW and TTC of 0.81 ± 0.08 mg/gDW. The product retains its light color, characteristic mild acrid taste and is consistent with the product's sensory properties.

## 4 CONCLUSION

We performed successful research on using technology solutions to utilize secondary raw materials with high nutritional value. The process of making jelly from cashew apples is perfected with good tannin removal which is acceptable in terms of product taste. The work done has retained the maximum nutritional content mainly in the processing processes such as blanching, osmosis, and drying. Cashew apple jelly kept TPC of 28.71%, TAA of 26.96%, and TTC of 27.3% compared to the starting material and recovery of 21%. The product not only has a big impact on the food market, but it also solves the problem of protecting the environment from organic industrial waste on a global scale.

## CONFLICT OF INTEREST

The authors declare that they have no conflicts of interest.

## AUTHOR CONTRIBUTION

**Tan Phat Dao:** Investigation (equal); Supervision (equal); Validation (equal); Writing – original draft (equal); Writing – review and editing (equal). **Vu Duong Nguyen:** Formal analysis (equal); Investigation (equal); Methodology (equal). **Ngoc Duc Vu:** Formal analysis (equal); Investigation (equal); Methodology (equal); Software (equal). **Thinh Van Pham:** Investigation (equal); Software (equal); Validation (equal); Visualization (equal). **Nhi Thi Yen Tran:** Formal analysis (equal); Investigation (equal); Methodology (equal); Software (equal); Validation (equal); Writing – original draft (equal).

## ETHICAL APPROVAL

This study does not involve any human or animal testing.

## Data Availability

Not applicable.

## References

[fsn32565-bib-0001] Abreu, F. P. , Dornier, M. , Dionisio, A. P. , Carail, M. , Caris‐Veyrat, C. , & Dhuique‐Mayer, C. (2013). Cashew apple (*Anacardium occidentale* L.) extract from by‐product of juice processing: A focus on carotenoids. Food Chemistry, 138(1), 25–31. 10.1016/j.foodchem.2012.10.028 23265451

[fsn32565-bib-0002] Argandoña, E. J. S. , Branco, I. G. , Takito, S. Y. , & Corbari, J. (2010). Influencia de la deshidratación osmótica y de la adición de cloruro de calcio en la conservación de kivis minimamente procesados. Food Science and Technology, 30, 205–209. 10.1590/S0101-20612010000500031

[fsn32565-bib-0003] Attri, B. L. (2009). Effect of initial sugar concentration on the physico‐chemical characteristics and sensory qualities of cashes apple wine. Natural Product Radiance, 8(4), 374–379.

[fsn32565-bib-0004] Bamidele, O. , Fasogbon, M. , Adebowale, O. , & Adeyanju, A. (2017). Effect of blanching time on total phenolic, antioxidant activities and mineral content of selected green leafy vegetables. Current Journal of Applied Science and Technology, 24(4), 1–8. 10.9734/CJAST/2017/34808

[fsn32565-bib-0005] Blanda, G. , Cerretani, L. , Cardinali, A. , Barbieri, S. , Bendini, A. , & Lercker, G. (2009). Osmotic dehydrofreezing of strawberries: Polyphenolic content, volatile profile and consumer acceptance. LWT – Food Science and Technology, 42(1), 30–36. 10.1016/j.lwt.2008.07.002

[fsn32565-bib-0006] Dao, T. P. , Nguyen, D. V. , Tran, T. Y. N. , Pham, T. N. , Nguyen, P. T. N. , Bach, L. G. , Nguyen, V. H. , Do, V. Q. , Nguyen, V. M. , & Tran, T. T. (2021). Effects of tannin, ascorbic acid, and total phenolic contents of cashew (*Anacardium occidentale* L.) apples blanched with saline solution. Food Research, 5(1), 409–416. 10.26656/fr.2017.5(1).454

[fsn32565-bib-0007] Das, I. , & Arora, A. (2017). Post‐harvest processing technology for cashew apple – A review. Journal of Food Engineering, 194, 87–98. 10.1016/j.jfoodeng.2016.09.011

[fsn32565-bib-0008] Deshpande, S. S. , Cheryan, M. , Salunkhe, D. K. , & Luh, B. S. (1986). Tannin analysis of food products. Critical Reviews in Food Science and Nutrition, 24(4), 401–449. 10.1080/10408398609527441 3536314

[fsn32565-bib-0009] Devic, E. , Guyot, S. , Daudin, J.‐D. , & Bonazzi, C. (2010). Effect of temperature and cultivar on polyphenol retention and mass transfer during osmotic dehydration of apples. Journal of Agricultural and Food Chemistry, 58(1), 606–614. 10.1021/jf903006g 19954217

[fsn32565-bib-0010] Dewanto, V. , Wu, X. , Adom, K. K. , & Liu, R. H. (2002). Thermal processing enhances the nutritional value of tomatoes by increasing total antioxidant activity. Journal of Agricultural and Food Chemistry, 50(10), 3010–3014. 10.1021/jf0115589 11982434

[fsn32565-bib-0011] Dimoso, N. , Makule, E. , & Kassim, N. (2020). Quality assessment of formulated osmotically dehydrated cashew apple (*Anacardium occidentale* L.) slices dried using hot air and solar driers. International Journal of Biosciences, 17(6), 421–432. 10.12692/ijb/17.6.421-432

[fsn32565-bib-0012] Emmanuelle, D. , Joseph, D. , Victor, A. , & Mohamed, M. S. (2016). A review of cashew (*Anacardiumoccidentale* L.) apple: Effects of processing techniques, properties and quality of juice. African Journal of Biotechnology, 15(47), 2637–2648. 10.5897/AJB2015.14974

[fsn32565-bib-0013] Francisco, M. , Velasco, P. , Moreno, D. A. , García‐Viguera, C. , & Cartea, M. E. (2010). Cooking methods of Brassica rapa affect the preservation of glucosinolates, phenolics and vitamin C. Food Research International, 43(5), 1455–1463. 10.1016/j.foodres.2010.04.024

[fsn32565-bib-0014] Gupta, S. , Jyothi, L. A. , & Prakash, J. (2008). Effect of different blanching treatments on ascorbic axit retention in green leafy vegetables. Natural Product Radiance, 7(2), 111–116.

[fsn32565-bib-0015] Johnston, C. S. , Corte, C. , & Swan, P. D. (2006). Marginal vitamin C status is associated with reduced fat oxidation during submaximal exercise in young adults. Nutrition & Metabolism, 3(1), 35. 10.1186/1743-7075-3-35 16945143 PMC1564400

[fsn32565-bib-0016] Lagnika, C. , Amoussa, A. M. O. , Sanni, A. , & Lagnika, L. (2019). Effect of blanching and ultrasound on drying time, physicochemical and bioactive compounds of dried cashew apple. American Journal of Food Science and Technology, 7(6), 227–233. 10.12691/ajfst-7-6-10

[fsn32565-bib-0017] Lavinas, F. C. , De Almeida, N. C. , Miguel, M. L. A. , Lopes, M. L. M. , & Valente‐Mesquita, V. L. (2006). Estudo da estabilidade química e microbiológica do suco de caju in natura armazenado em diferentes condições de estocagem. Ciencia E Tecnologia De Alimentos, 26(4), 875–883. 10.1590/S0101-20612006000400026

[fsn32565-bib-0018] Najafi, A. H. , Yusof, A. Y. , Rahman, A. R. , Ganjloo, A. , & Ling, N. C. (2014). Effect of osmotic dehyration process using sucrose solution at mild temperature on mass tranffer and quality attributes of red pitaya (*Hylocereus polyrhizusis*). International Food Research Journal, 21(2), 625–630.

[fsn32565-bib-0019] Nguyen, N. Q. , Nguyen, M. T. , Nguyen, V. T. , Le, V. M. , Trieu, L. H. , Le, X. T. , Khang, T. V. , Giang, N. T. L. , Thach, N. Q. , & Hung, T. T. (2020). The effects of different extraction conditions on the polyphenol, flavonoids components and antioxidant activity of *Polyscias fruticosa* roots. IOP Conference Series: Materials Science and Engineering, 736, 349–354. 10.1088/1757-899X/736/2/022067

[fsn32565-bib-0020] Nguyen, T. V. L. , Tran, T. Y. N. , Lam, D. T. , Bach, L. G. , & Nguyen, D. C. (2019). Effects of microwave blanching conditions on the quality of green asparagus (*Asparagus officinalis* L.) butt segment. Food Science & Nutrition, 7(11), 3513–3519. 10.1002/fsn3.1199 31763001 PMC6848841

[fsn32565-bib-0021] Nhi, T. T. Y. , Thinh, P. V. , Vu, N. D. , Bay, N. T. , Tho, N. T. M. , Quyen, N. N. , & Truc, T. T. (2020). Kinetic model of moisture diffusivity in soursop leaves (*Annona muricata L*.) by convection drying. IOP Conference Series: Materials Science and Engineering, 991, e012107. 10.1088/1757-899X/991/1/012107

[fsn32565-bib-0022] Olalusi, A. P. , & Erinle, O. (2019). Influence of drying temperature and pretreatment on the drying characteristics and quality of dried cashew (*Anacardium occidentale* L.) apple slices. *Croatian* . Journal of Food Science and Technology, 11(1), 97–103. 10.17508/CJFST.2019.11.1.14

[fsn32565-bib-0023] Pham, T. N. , Nguyen, V. T. , Toan, T. Q. , Cang, M. H. , Bach, L. G. , & Van Muoi, N. (2020). Effects of various processing parameters on polyphenols, flavonoids, and antioxidant activities of *Codonopsis javanica* root extract. Natural Product Communications, 15(9), 1934578X2095327. 10.1177/1934578X20953276

[fsn32565-bib-0024] Prathapan, A. , Lukhman, M. , Arumughan, C. , Sundaresan, A. , & Raghu, K. G. (2009). Effect of heat treatment on curcuminoid, colour value and total polyphenols of fresh turmeric rhizome. International Journal of Food Science & Technology, 44(7), 1438–1444. 10.1111/j.1365-2621.2009.01976.x

[fsn32565-bib-0025] Salehi, B. , Özgüven, M. G. , Kirkin, C. , Özçelik, B. , Braga, M. F. B. M. , Carneiro, J. N. P. , Bezerra, C. F. , Silva, T. G. , Coutinho, H. D. M. , Amina, B. , Armstrong, L. , Selamoglu, Z. , Sevindik, M. , Yousaf, Z. , Rad, J. S. , Muddathir, A. M. , Devkota, H. P. , Martorell, M. , Jugran, A. K. , & Martins, N. (2020). Antioxidant, antimicrobial, and anticancer effects of anacardium plants: An ethnopharmacological perspective. Frontiers in Endocrinology, 11, 295. 10.3389/fendo.2020.00295 32595597 PMC7303264

[fsn32565-bib-0026] Sereno, A. M. , Moreira, R. , & Martinez, E. (2001). Mass transfer coefficients during osmotic dehydration of apple in single and combined aqueous solutions of sugar and salt. Journal of Food Engineering, 47(1), 43–49. 10.1016/S0260-8774(00)00098-4

[fsn32565-bib-0027] Sieniawska, E. , & Baj, T. (2017). Tannins. Dans Pharmacognosy (pp. 199–232). Elsevier. 10.1016/B978-0-12-802104-0.00010-X

[fsn32565-bib-0028] Talasila, U. , Vechalapu, R. R. , & Shaik, K. B. (2012). Clarification, preservation, and shelf life evaluation of cashew apple juice. Food Science and Biotechnology, 21(3), 709–714. 10.1007/s10068-012-0092-3

[fsn32565-bib-0029] Tran, N. Y. T. , Nhan, N. P. T. , Thanh, V. T. , Chinh, N. D. , Tri, D. L. , Nguyen, D. V. , Vy, T. A. , Truc, T. T. , & Thinh, P. V. (2020). Effect of storage condition on color, vitamin C content, polyphenol content and antioxidant activity in fresh soursop pulp (*Annona muricata* L.). IOP Conference Series: Materials Science and Engineering, 736, e022065. 10.1088/1757-899X/736/2/022065

[fsn32565-bib-0030] von Assis, A. V. R. D. , Bizzo, H. R. , Matta, V. M. D. , & Cabral, L. M. C. (2007). Recuperação e concentração de componentes do aroma de caju (*Anacardium occidentale* L.) por pervaporação. Ciência E Tecnologia De Alimentos, 27(2), 349–354. 10.1590/S0101-20612007000200024

